# Post-mission debriefs in helicopter emergency medicine services– introducing “The compassionate debrief”

**DOI:** 10.1186/s13049-025-01326-1

**Published:** 2025-01-24

**Authors:** Leif Rognås, Marius Rehn, Karina Damsgaard Nørby

**Affiliations:** 1 Danish Air Ambulance, Brendstrupgårdsvej 7, 2th, 8200 Aarhus N, Denmark; 2 Department of Anaesthesia, Aarhus University Hospital, Palle Juul-Jensens Boulevard 99, 8200 Aarhus N, Denmark; 3 Air Ambulance Department, Division of Prehospital Services, Oslo University Hospital, Oslo, Norway; 4 Institute of Clinical Medicine, University of Oslo, Blindern, Odense, Postbox 1072, Denmark; 5 Department of Research and Development, Norwegian Air Ambulance Foundation, Oslo, Norway; 6 Department of Regional Health Research, University of Southern Denmark, Odense, Denmark; 7 Corporate HR, Central Denmark Region, Olof Palmes Allé 32, 8200 Aarhus N, Denmark; 8 Department of Clinical Medicine, Aarhus University, Palle Juul-Jensens Boulevard 11, 8200 Aarhus N, Denmark

## Status of the post-mission debriefs in helicopter emergency medical services (HEMS)

Rounding up clinical HEMS missions with a structured discussion is routine in many high-functioning organisations.

An international group of experts included the question “*Was the mission debriefed*?” as a process indicator when they in 2017 suggested a standard set of quality indicators (QI) for physician-staffed emergency medical services (P-EMS) [1].

In 2019, Haugland et al. tested these QI in a prospective multicentre study involving 16 Nordic P-EMS and found that the percentage of missions debriefed was a QI with good feasibility, rankability, variability and actionability [2].

In a third study however, the same group found that in Nordic P-EMS, this QI was not associated with any difference in 30-day mortality [3] although the effect on mortality may not be the best way to measure the effect of a debrief as it does not take into account the potential effects on for instance on-scene collaborations and communications.

## The “honest” debrief in HEMS

HEMS crews often conduct post-mission debriefs following scripts that are slightly adapted versions of a post-simulation or post-training scenario debrief. These debriefs, which sometimes include members of the wider team (i.e. dispatchers), are often referred to as “honest” debriefs.

The term “honest debrief” has a rather aloof origin but is frequently used and promoted in settings where high-performing teams meet up after a training session, a job, or a mission. The focus of these debriefs is often performance enhancement through an open and direct conversation and they are typically led by a trained instructor, a trainer / coach, a facilitator, or a senior staff member who often has a set of learning points that she wants to get across. Typically, there is an implicit understanding, both between the leader and the team and within the team, that the purpose is to evaluate the team’s performance, with the debrief leader having the final say in this evaluation. This means that there is a predefined and clear hierarchy or “knowledge gradient” between the person leading the debrief and the team being debriefed.

## Why the current way of conducting HEMS post-mission debriefs may not be ideal

If done sensibly, with kindness and consideration, the post-mission debrief is a tremendous learning and development opportunity. We do feel however, that there are several problems with how post-mission debriefs are conducted in many HEMS:

- In most cases, HEMS crews conduct their own debriefs, as they are the only ones on base 24/7/365. These teams are characterised by a flat hierarchy with no overall leader; in-flight, the captain is the leader, the HEMS doctor leads on patient evaluation, treatment and triage and the HEMS paramedic leads on logistics, on-scene tactics and extrication.

So, who is then going to lead the debrief? What does the knowledge gradient look like? Is there even a knowledge- or command gradient? How does this influence the debrief?

- The concept of the honest debrief tends to assume that there is one correct or preferred way of doing things. in contrast, HEMS very often operate within shades of grey with no definitive rights and wrongs:

Was it “correct” to transfuse this patient?

Was it “wrong” not to take this patient to a Major Trauma Centre?

Was it “correct” to land in the terrain instead of at a pre-surveyed site?

There are rarely clear-cut answers to such questions and just because the colleagues we worked with last week chose to solve a similar case differently does not mean that they were wrong, nor does it mean that we were wrong, just that jobs, crews and settings are different.

So, how does this affect the debrief, our feedback to colleagues, and our learning points? And perhaps even more importantly; how should it influence the way we conduct our debriefs?

## How the “honest debrief” may affect us

If we in our post-mission debriefs use scripts, phraseology, and mindsets more suited for debriefing moulages or training sessions, we worry that we may do more harm than good.

Having worked in various HEMS / P-EMS organisations in several different countries, hearing colleagues say, “*The debrief felt like doctor bashing*”, “*They tore me to pieces during that debrief*” and “*Following that debrief*,* I had a knot in my stomach for the rest of the weekend*” is, sadly, not unfamiliar to us.

There is, of course, the important but relatively rare need to identify, discus, and correct clear-cut mistakes. However, testimonials like the ones above have made us question whether the honest debrief truly allows us to treat our crew mates - and ourselves - in a way that fosters a sense of belonging, acceptance, and respect; all of which are essential for optimising learning and growth.

In addition, it is hard to imagine how stress, moral injury, and a sense of lack of control can be addressed if mutual trust is not established.

## Could compassion be part of the solution?

People new to the scientific concept of compassion often ask about the difference between compassion and empathy. Professor Paul Gilbert, the psychologist who introduced the term, famously explained that empathy is a skill, whereas compassion is an intent; the intent to be sensitive to suffering of oneself and others, with a commitment to alleviate and prevent it.

A contemporary review of how compassion may impact patient safety and quality in health care is provided by Ahmed et al. [4].

## The compassionate post-mission debrief

We suggest that HEMS organisations may benefit from exploring the possibility of adjusting their post-mission debriefs so that crew members approach them with the following questions in mind:

- How can I be helpful, not hurtful?
- Am I giving my crew mates and myself the benefit of the doubt?
- What do we as individuals and the organisation as a whole need, both now and in the longer run?

To our knowledge, we are the first to use the term “compassionate debrief”.

We believe we owe it to our patients, our colleagues, and ourselves to do better, and to do that, we need to carefully evaluate our debriefs and their impact. Implementing these changes will take effort, deliberate practice, and multidisciplinary research, but may improve staff well-being and enhance learning outcomes.

## Data Availability

No datasets were generated or analysed during the current study.
